# Cardiac autonomic profile, perceived stress and environmental comfort in healthy employees during remote and in-office work

**DOI:** 10.1038/s41598-024-54283-7

**Published:** 2024-02-14

**Authors:** Francesca Perego, Beatrice De Maria, Monica Parati, Giuseppina Cassetti, Alessandra Gorini, Vlasta Bari, Francesca Gelpi, Alberto Porta, Laura Adelaide Dalla Vecchia

**Affiliations:** 1 Istituti Clinici Scientifici Maugeri IRCCS, Via Camaldoli 64, 20138 Milan, Italy; 2 Dipartimento di Scienze Cliniche e di Comunità, Università degli Studi di Milano, Via Della Commenda 19, 20122 Milan, Italy; 3 Department of Biomedical Sciences for Health, University of Milan, Via Mangiagalli 31, 20133 Milan, Italy; 4 Department of Cardiothoracic, Vascular Anesthesia and Intensive Care, IRCCS Policlinico San Donato, Piazza Edmondo Malan 2, San Donato Milanese, 20097 Milan, Italy

**Keywords:** Remote working, Autonomic nervous system, Heart rate variability, Cardiovascular prevention, Stress, Cardiology, Risk factors

## Abstract

Remote work (REMOTE) causes an overlap between working and domestic demands. The study of the cardiac autonomic profile (CAP) by means of heart rate variability (HRV) provides information about the impact of REMOTE on workers’ health. The primary aim was to determine whether CAP, self-perceived stress, environmental and workstation comfort are modified during REMOTE. The secondary aim was to explore how these indices are influenced by individual and environmental work-related factors. Fifty healthy office employees alternating REMOTE and in-office (OFFICE) working were enrolled, rated self-perceived stress, environmental and workstation comfort using a visual analogue scale and performed a 24-h electrocardiogram during REMOTE and OFFICE. Stress was lower (5.6 ± 2.2 vs. 6.4 ± 1.8), environmental comfort higher (7.7 ± 1.9 vs. 7.0 ± 1.5), and the workstation comfort poorer (6.2 ± 1.8 vs. 7.5 ± 1.2) during REMOTE. CAP was similar during REMOTE and OFFICE. CAP was influenced by some work-related factors, including the presence of offspring, absence of a dedicated workspace during REMOTE and number of working hours. All these variables determined a decreased vagal modulation. The working setting seems to impact the levels of perceived stress and comfort, but not the CAP. However, individual and environmental work-related factors reduce cardiac vagal modulation during REMOTE, potentially increasing the risk of developing cardiovascular diseases.

## Introduction

Remote work has revolutionized the way of thinking and living in the working environment extending it to a virtually infinite space, removing the obligatory physical wall of a confined setting, and often modifying the temporal rigidity of the amount of time devoted to work^1–3^. Remote work also blurred the lines between working and personal demands. It changed familial relationships, physical activity and eating habits, household demands, work-related stress, and productivity, and potentially introduced new health-related risk factors^4–9^. In particular, overwork, long working hours, and the tendency of many workers to stay connected at any time every day of the week are known to be potential risk factors for cardiovascular diseases^10–12^. Similarly, remote work seems to have negative effects on the employees’ psychological well-being mainly due to an increased conflict between private and working life, with a consequent increase in stress and discomfort^13,14^. However, to date, there are no studies that investigate whether these effects are related to changes at a physiological level, such as cardiac neural regulation^15^.

The study of the cardiac autonomic profile (CAP) can be performed by an easy and non-invasive recording of the electrocardiogram (ECG) for a period ranging from 5 min to 24 h^16,17^. In particular, the analysis of the continuous ECG by means of the heart rate variability (HRV) techniques provides quantitative indices of sympathetic and vagal modulation contributing to the CAP. These markers are largely accepted for the definition of the pathophysiology and prognosis of several diseases, including hypertension, myocardial infarction, heart failure, and physiological conditions, such as exercise, stressing challenges, and ageing^18–22^.

**Figure 1:**
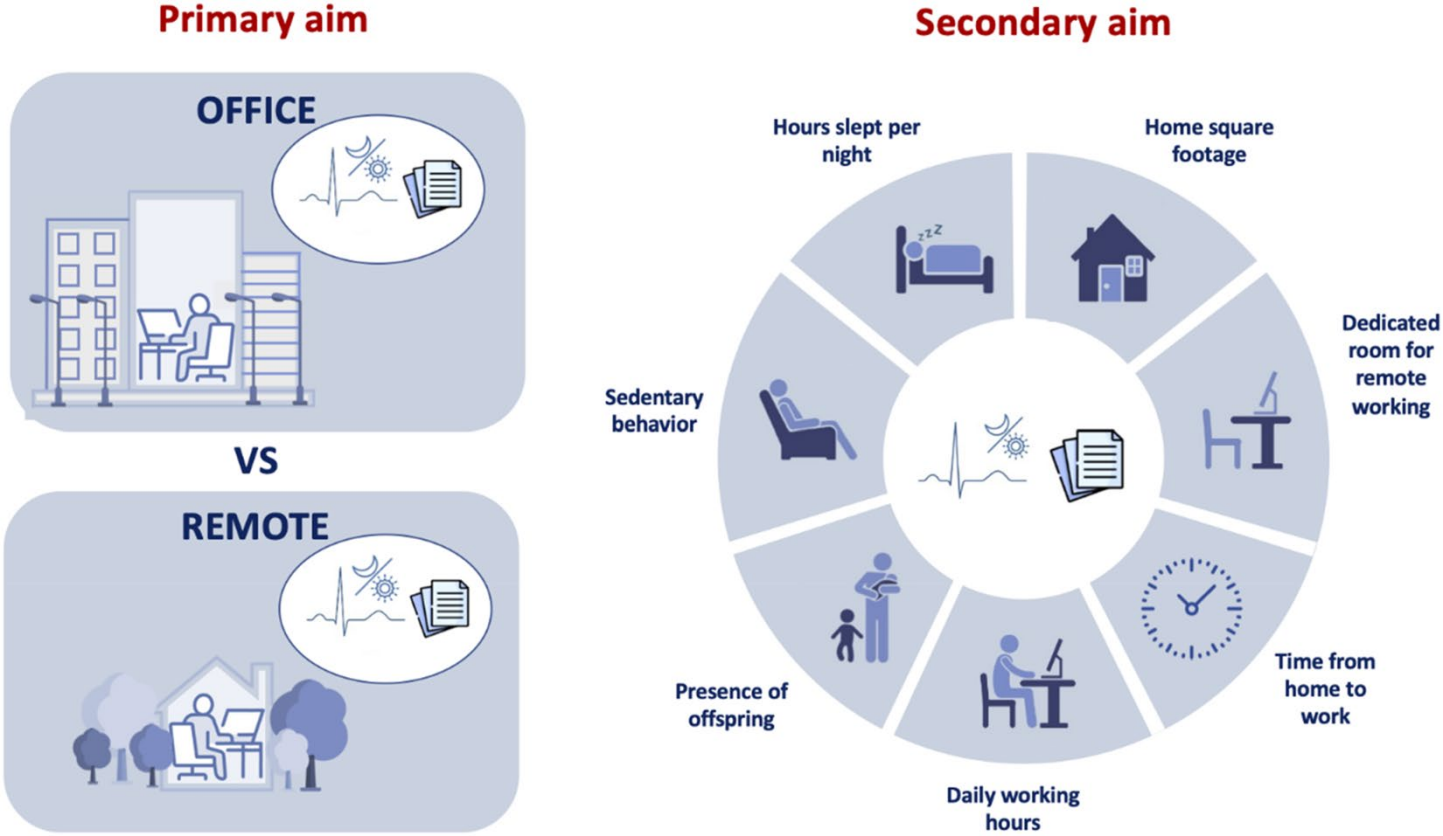
Schematic representation of the experimental protocol. Left panel-primary aim: comparison between remote versus in-office working in terms of diurnal and nocturnal cardiac autonomic profile, perceived stress, environmental and workstation comfort. Right panel-secondary aim: correlation between the variables considered in the primary aim and the objective factors related to individual or environmental conditions (created with biorender.com).

The primary aim of this study was to compare remote versus in-office working in terms of differences in: (i) diurnal and nocturnal CAP, (ii) perceived stress, (iii) perceived environmental comfort, (iv) perceived workstation comfort, and to assess possible correlations between these variables (Fig. 1, left panel). The secondary aim was to correlate the four above-mentioned variables with objective factors related to individual or environmental work-related conditions (7 variables, see Fig. 1, right panel).

## Methods

### Population and experimental protocol

Office employees, alternating days of work at home (REMOTE) and in the office (OFFICE) during the same week, were enrolled.

The ratio between male and female subjects was fixed at 1:1. The study protocol was completed in the period from July 2020 to July 2021. The sample size of the study was calculated based on previous studies on similar topics^23,24^ and was fixed at 50 subjects.

The inclusion criteria were: (i) age higher than 18 years; (ii) healthy subjects; (iii) active full-time workers. The exclusion criteria were: (i) use of any regular medication; (ii) heavy smokers^25^; (iii) heavy alcohol drinkers^26^.

At the enrollment, detailed demographic and anamnestic data were collected to characterize the participants’ sample.

A 3-lead 24-h Holter ECG (360° eMotion FAROS, MegaElectronics, Finland; Sylco Srl, Monza, Italy) was acquired at a sampling rate of 500 Hz during REMOTE and OFFICE days, in a randomized order. In addition, perceived stress, environmental comfort, and workplace comfort were collected through self-administered assessments during both OFFICE and REMOTE days. CAP data were analysed during day-time (DAY) and night-time (NIGHT). DAY was sampled from 1 to 4 pm, a time period when all participants were working. NIGHT was sampled in a time period from 1 to 4 am when all participants reported sleeping.

Seven variables related to individual or environmental conditions factors were also collected during OFFICE and REMOTE days by self-administered ad-hoc questionnaire.

The study adhered to the principles of the Declaration of Helsinki for medical research involving human subjects. All the participants signed a written informed consent and the protocol was approved by the Istituti Clinici Scientifici Maugeri Ethics Committee (2467CE).

Preliminary results of the study have been presented at conferences^27,28^.

### Cardiac autonomic profile characterization

Lead II of the ECG was chosen for analysis, due to the best signal-to-noise ratio. The RR interval time, i.e. the time distance between two consecutive R peaks on the ECG was derived by means of an automatic algorithm^23,24^. Detections of R wave peaks were manually checked to avoid bias due to misdetections. Correction of ectopic beats was performed by cubic spline interpolation, never exceeding 5% of total beats.

Over each derived RR series, segments of 5000 consecutive RR intervals were selected during DAY and NIGHT for further analysis. On these selections, an iterated analysis on windows of 250 consecutive RR intervals, with a superimposition of 200, was performed^29^. The median of the whole distribution was taken as representative of the entire series. The mean (μ_RR_) and variance (σ^2^_RR_) of RR intervals were computed and expressed in ms and ms^2^, respectively.

To complete the characterization of the CAP, autoregressive power spectral decomposition was applied to the RR series after optimizing the model order with the Akaike information criterion. The sum of the power spectral components whose central frequency dropped in the range of the high frequency (HF) band (0.15–0.40 Hz) was labelled as HF_RR_, expressed in absolute units (i.e. ms^2^) and taken as an index of the cardiac vagal modulation directed to the heart. This index is recognized to be solid to describe and furnish a straightforward interpretation of vagal cardiac modulation^17^. HF_RR_ was also expressed in normalized units and labelled as HF_RRnu_^30,31^.

### Measure of perceived stress, indoor environmental comfort, and workstation comfort

Participants were asked to self-report their perceived level of stress on a Visual Analogue Scale (VAS), represented by a 100 mm horizontal line where the minimum score (i.e. 0) indicated “no perceived stress”, and the maximum score (i.e. 10) indicated a “very high perceived stress”^32,33^. The participants were asked to mark the line prompted by the following “*Please mark your average stress level during working”.* Participants were then instructed to assess their self-perceived indoor environmental comfort (“*Please mark your average comfort level related to the indoor work environment, in terms of lighting, temperature, ventilation, air quality, noise*”) as well as their workstation comfort (*“Please mark your average comfort level related to the workstation, in terms of sitting posture, desk size, position of the monitor and keyboard”)* on two additional VAS where the extreme items indicated “very low comfort” (i.e. 0) and “very high comfort” (i.e. 10), respectively^34–37^. Such measurements were performed both in REMOTE and OFFICE.

### Individual and environmental work-related factors

A self-administered ad hoc survey was provided to the participants to obtain some information regarding individual and environmental work-related factors. In detail, the number of hours slept per night, the number of daily working hours, and the home square footage were collected as continuous variables, while the presence of offspring, the time from home to work (more or less than 30 min), the presence of a dedicated room when REMOTE, and whether they had a sedentary behaviour were recorded as dichotomous variables.

### Statistical analysis

Continuous data were reported as mean ± standard deviation and dichotomous data were reported as absolute values and percentages. To characterize the participants’ sample and provide a description of their habits during the two different working conditions, the comparison of individual and environmental work-related variables in REMOTE and OFFICE was performed. Paired t-test or Wilcoxon signed rank test in case of not-normal distribution was accordingly applied for continuous data. For categorical variables, the χ^2^ test was utilized. Shapiro–Wilk test was applied to test the normality of the data.

To answer the primary aim, the two-way repeated measures analysis of variance (two-factor repetition, Holm-Sidak test for multiple comparisons) was performed to check the differences of the CAP indices between the two experimental conditions (i.e. REMOTE and OFFICE) within the same period of analysis (i.e. DAY or NIGHT), and between periods of analysis within the same working condition. In addition, a paired t-test or Wilcoxon signed rank test in the case of non-normal distribution was applied to examine possible differences between REMOTE and OFFICE in perceived stress, indoor environmental quality, and workstation comfort. The correlations between nocturnal CAP indices and perceived stress, indoor environmental quality, and workstation comfort were additionally investigated using Spearman rank correlation coefficient ρ and the probability p of a type I error.

To answer the secondary aim, two different statistical analyses were applied based on the continuous or dichotomous nature of the collected individual and environmental work-related variables. In the case of a dichotomous variable, the population data related to the CAP, self-perceived stress, environmental comfort, and workstation comfort were split into two groups and compared. In detail, a two-way repeated measures analysis of variance (two-factor repetition, Holm-Sidak test for multiple comparisons) was applied to examine the influence of dichotomous variables (i.e. presence of children, sedentary behaviour, presence of a dedicated room during REMOTE, time to reach the workplace greater than 30 min) on CAP indices and self-perceived stress, indoor environmental comfort, and workstation comfort in the two experimental conditions (REMOTE and OFFICE). Instead, correlation analysis by means of Spearman rank correlation coefficient ρ and the probability p of a type I error was performed to examine possible associations between the continuous work-related factors (i.e. slept hours per night, working hours per day, dimension of the house) and the nocturnal CAP indices and the self-perception of stress, indoor environmental quality and workstation comfort. We conducted correlation analyses during night-time because there is recent evidence that CAP differences in healthy subjects are more evident during night-time, a more standardized period as subjects are sleeping^23,24^.

A p < 0.05 was always considered significant. Statistical analyses were carried out using Sigmaplot, Systat Software, Inc., Chicago, IL, version 11.0.

## Results

### Description of the enrolled population

Fifty healthy office employees (25 males and 25 females, mean age 39 ± 11 years, body mass index 23.4 ± 3.5 kg·m^2^) were enrolled in the study. Their occupations spanned different fields including information technology (48%), business and administration (18%), health sciences (18%), law (6%), environmental sciences (6%), and architecture (4%). Most subjects (n = 42, 84%) had a permanent employment contract, and 96% (n = 48) had been working for more than 1 year. Ten subjects (20%) were light smokers and 27 (54%) were social drinkers. Nineteen (38%) subjects were sedentary, i.e. they did not perform any regular aerobic or anaerobic physical activity. The majority (n = 44, 88%) of subjects had at least one cohabitant, 23 (46%) had offspring and 9 (18%) had pets. The home square footage was 98 ± 33 m^2^. Thirty-two (64%) subjects reported having a dedicated room during REMOTE.

Comparing habits between REMOTE and OFFICE, the employees slept more (7.3 ± 0.8 vs. 6.9 ± 0.8 h per night, p < 0.05) and worked longer (8.7 ± 1.3 vs. 8.4 ± 1.0 h per day, p < 0.05) during REMOTE, compared to OFFICE. As to the physical activity, considering only the active subjects (n = 30, 60%), 26 of them (87%) exercised equally in REMOTE and OFFICE days, 1 only in OFFICE days, and 3 only in REMOTE days. The mean weekly hours dedicated to physical activity during REMOTE and OFFICE were similar (3.1 ± 1.7 vs. 3.1 ± 2.2 h).

### Primary aim: results of the comparison between CAP, perceived stress, work station and environmental comfort between REMOTE and OFFICE

Table 1 shows the results of the comparison of CAP indices in REMOTE and OFFICE, during DAY and NIGHT, as well as the overall results of the comparison of perceived stress, environmental and workstation comfort in REMOTE and OFFICE.

**Table 1:** Comparison of CAP indices, self-perceived stress, environmental comfort, and workstation comfort between a day of remote working (REMOTE) and a day at office (OFFICE).

	REMOTE		OFFICE	
	DAY	NIGHT	DAY	NIGHT
µ_RR_ [ms]	838.9 ± 129.6	988.1 ± 140.8*	819.8 ± 124.3	997.6 ± 150.0*
σ^2^_RR_ [ms^2^]	4162.0 ± 3466.2	4917.3 ± 4909.2	3933.1 ± 2854.8	5063.4 ± 5266.4
HF_RR_ [ms^2^]	459.6 ± 808.1	1118.0 ± 1771.2*	378.1 ± 444.6	1188.2 ± 1674.1*
HF_RRnu_ [nu]	25.7 ± 16.0	43.1 ± 18.5*	22.4 ± 11.1	45.0 ± 17.4*
Perceived stress [0–10]	5.6 ± 2.2		6.4 ± 1.8^#^	
Environmental comfort [0–10]	7.7 ± 1.9		7.0 ± 1.5#	
Workstation comfort [0–10]	6.2 ± 1.8		7.5 ± 1.2^#^	

REMOTE, working day in remote working; OFFICE, working day in the office; RR, RR interval; µ_RR_, mean RR; σ^2^_RR_, variance of RR; HF, high frequency; HF_RR_, power of RR in the HF band; HF_RRnu_, normalized power of RR in the HF band. Data are presented as mean ± standard deviation.

*Indicates p < 0.05 DAY vs. NIGHT.

^#^Indicates p < 0.05 REMOTE vs. OFFICE.

µ_RR_, HF_RR_, and HF_RRnu_ increased from DAY to NIGHT in both working conditions (REMOTE and OFFICE). σ^2^_RR_ was unchanged between DAY and NIGHT in both working conditions. No differences between REMOTE and OFFICE were detected for any of the considered indices (i.e. µ_RR_, σ^2^_RR_, HF_RR_, and HF_RRnu_). The perceived stress was lower during REMOTE and the perceived indoor environmental comfort was higher in REMOTE, although the workstation comfort was better in OFFICE.

All nocturnal CAP indices were found to be uncorrelated to perceived stress, indoor environmental comfort, and workstation comfort in both REMOTE and OFFICE, except HF_RR_ and perceived indoor environmental comfort, which were significantly correlated: the higher the environmental comfort, the higher the HF_RR_, as shown in Table 2.

**Table 2:** Results of the correlation analysis between the nocturnal CAP indices, perceived stress, environmental and workstation comfort in REMOTE and OFFICE.

	µ_RR_ [ms]		σ^2^_RR_ [ms^2^]		HF_RR_ [ms^2^]		HF_RRnu_ [nu]	
	*ρ*	*p*	*ρ*	*p*	*ρ*	*p*	*ρ*	*p*
REMOTE								
Perceived stress [0–10]	− 0.173	0.234	− 0.231	0.111	− 0.148	0.308	0.026	0.856
Environmental comfort [0–10]	0.236	0.102	0.201	0.166	**0.254**	**0.048**	0.200	0.167
Workstation comfort [0–10]	0.108	0.459	0.080	0.583	− 0.011	0.937	0.007	0.960
OFFICE								
Perceived stress [0–10]	− 0.052	0.723	0.047	0.747	0.002	0.987	0.056	0.703
Environmental comfort [0–10]	− 0.056	0.700	0.080	0.584	0.073	0.617	− 0.077	0.597
Workstation comfort [0–10]	− 0.025	0.862	0.149	0.304	0.170	0.241	− 0.176	0.225

Significant values are in bold.

REMOTE, working day in remote working; OFFICE, working day in the office; RR, RR interval; µ_RR_, mean RR; σ^2^_RR_, variance of RR; HF, high frequency; HF_RR_, power of RR in the HF band; HF_RRnu_, normalized power of RR in the HF band; *ρ* Spearman rank correlation coefficient; p, p value.

### Secondary aim: association of CAP indices, self-perceived stress, indoor environmental comfort and workstation comfort with individual and environmental work-related factors

The correlation analyses between nocturnal CAP indices, perceived stress, indoor environmental comfort, workstation comfort, working hours per day, slept hours per night, and the home square footage are shown in Table 3, separately for OFFICE and REMOTE.

**Table 3:** Results of the correlation analysis between nocturnal CAP indices, perceived stress, comfort and work-related factors in REMOTE and OFFICE.

	µ_RR_ [ms]		σ^2^_RR_ [ms^2^]		HF_RR_ [ms^2^]		HF_RRnu_ [nu]		Perceived stress [0–10]		Workstation comfort [0–10]		Environmental comfort [0–10]	
	*ρ*	*p*	*ρ*	*p*	*ρ*	*p*	*ρ*	*p*	*ρ*	*p*	*ρ*	*p*	*ρ*	*p*
REMOTE														
Working hours per day, hours	− **0.330**	**0.021**	− **0.355**	**0.012**	− **0.364**	**0.010**	− **0.395**	**0.005**	**0.336**	**0.019**	− 0.052	0.723	− 0.082	0.572
Slept hours per night, hours	0.123	0.409	0.238	0.106	0.275	0.062	0.125	0.400	− 0.149	0.315	**0.378**	**0.009**	0.022	0.883
Dimension of the house, m^2^	0.108	0.470	− 0.184	0.213	− 0.207	0.162	0.014	0.922	− 0.007	0.964	− 0.057	0.700	− 0.130	0.383
OFFICE														
Working hours per day, hours	− 0.152	0.301	− 0.075	0.612	− 0.255	0.080	− **0.409**	**0.004**	0.273	0.061	0.278	0.056	**0.317**	**0.028**
Slept hours per night, hours	0.050	0.741	0.097	0.521	0.026	0.864	− 0.156	0.299	− 0.174	0.248	0.274	0.065	0.153	0.309
Dimension of the house, m^2^	0.273	0.063	0.136	0.360	− 0.066	0.658	− 0.141	0.343	− 0.208	0.160	− 0.107	0.472	0.038	0.800

Significant values are in bold.

REMOTE, working day in remote working; OFFICE, working day in the office; RR, RR interval; µ_RR_, mean RR; σ^2^_RR_, variance of RR; HF, high frequency; HF_RR_, power of RR in the HF band; HF_RRnu_, normalized power of RR in the HF band; *ρ* Spearman rank correlation coefficient; p, p value.

A negative correlation between working hours per day and all the CAP indices was observed in REMOTE, while during OFFICE it was only noticed for the HF_RRnu_.

A positive correlation between HF_RR_ and the reported indoor environmental comfort was found during REMOTE, suggesting that a higher perceived environmental comfort is associated with a higher nocturnal vagal modulation to the sinus node. No other significant correlations were found.

**Figure 2:**
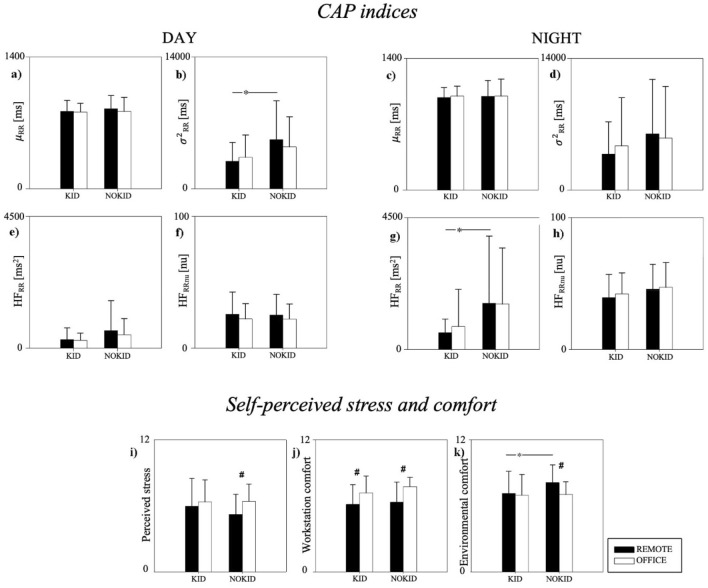
Comparison of CAP indices (panels (**a**–**h**)), self-perceived stress (panel (**i**)), environmental comfort (panel (**j**)) and workstation comfort (panel (**k**)) between REMOTE and OFFICE stratifying the population according to the presence (KID) or absence (NOKID) of children. CAP indices were compared during daytime (DAY, panels (**a**,**b**,**e**,**f**)) and during night-time (NIGHT, panels (**c**,**d**,**g**,**h**)). Results are presented as mean ± standard deviation. *Indicates p < 0.05 DAY vs. NIGHT. ^#^Indicates p < 0.05 REMOTE vs. OFFICE.

To study the impact of children on the study variables, the population was divided into two groups according to their presence (KID group, n = 23, age 48 ± 8 years, 9 males and 14 females) or absence (NOKID group, n = 27, age 32 ± 6 years, 16 males and 11 females). In the KID group, 9 subjects had only one child, 11 had two children, and 3 had three children. The ages of the children ranged from 1 to 16 years. The results are reported in Fig. 2. The σ^2^_RR_ was higher in the NOKID group compared to the KID group during DAY, in REMOTE. The HF_RR_ was higher in the NOKID group compared to the KID group during NIGHT, in REMOTE. No other significant differences between REMOTE and OFFICE were detected for µ_RR_, HF_RR_, and HF_RRnu_ during DAY and for µ_RR_, σ^2^_RR_, and HF_RRnu_ during NIGHT. The NOKID group reported being more stressed while working in OFFICE than in REMOTE. No significant differences between REMOTE and OFFICE were noticed in perceived stress in the KID group. Both KID and NOKID groups conveyed that the workstation comfort was better at OFFICE than at REMOTE, whereas the NOKID group only found that the indoor environmental comfort was lower at OFFICE than at REMOTE.

**Figure 3:**
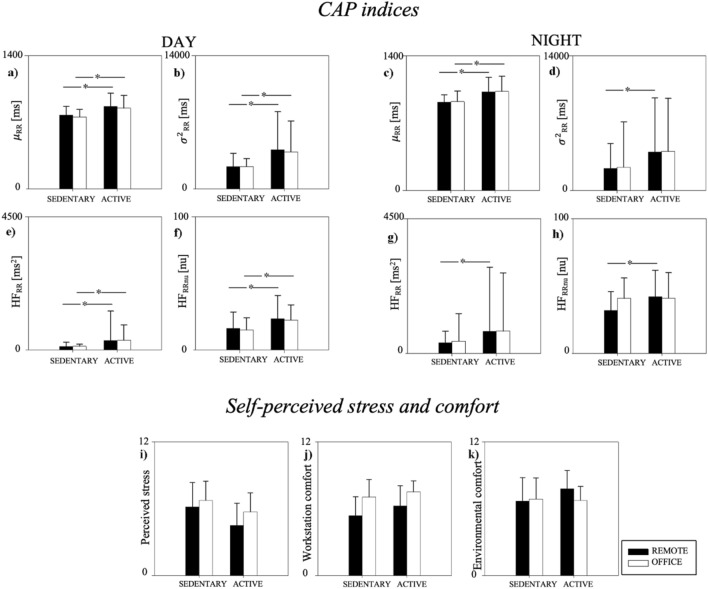
Comparison of CAP indices (panels (**a**–**h**)), self-perceived stress (panel (**i**)), environmental comfort (panel (**j**)) and workstation comfort (panel (**k**)) between REMOTE and OFFICE stratifying the population based on absence (SEDENTARY) or presence (ACTIVE) of any regular aerobic or anaerobic physical activity. CAP indices were compared during day-time (DAY, panels (**a**,**b**,**e**,**f**)) and during night-time (NIGHT, panels (**c**,**d**,**g**,**h**)). Results are presented as mean ± standard deviation. *Indicates p < 0.05 DAY vs NIGHT. ^#^Indicates p < 0.05 REMOTE vs. OFFICE.

Figure 3 displays the comparison of indices related to CAP, stress, environmental comfort, and workstation comfort between sedentary (SEDENTARY group, n = 20, age 42 ± 10 years, 9 males and 11 females) and non-sedentary subjects (ACTIVE group, n = 30, age 37 ± 11 years, 16 males and 14 females). During DAY, CAP indices were higher in the ACTIVE group than in the SEDENTARY one, while they were not different between REMOTE and OFFICE. During NIGHT, µ_RR_ was higher in the ACTIVE group than the SEDENTARY group, both in REMOTE and OFFICE. Differences in σ^2^_RR_, HF_RR_, and HF_RRnu_ were also noticed between the SEDENTARY and ACTIVE groups during REMOTE. No relevant changes between the SEDENTARY and ACTIVE groups were noticed for the perceived stress, indoor environmental comfort, and workstation comfort.

**Figure 4:**
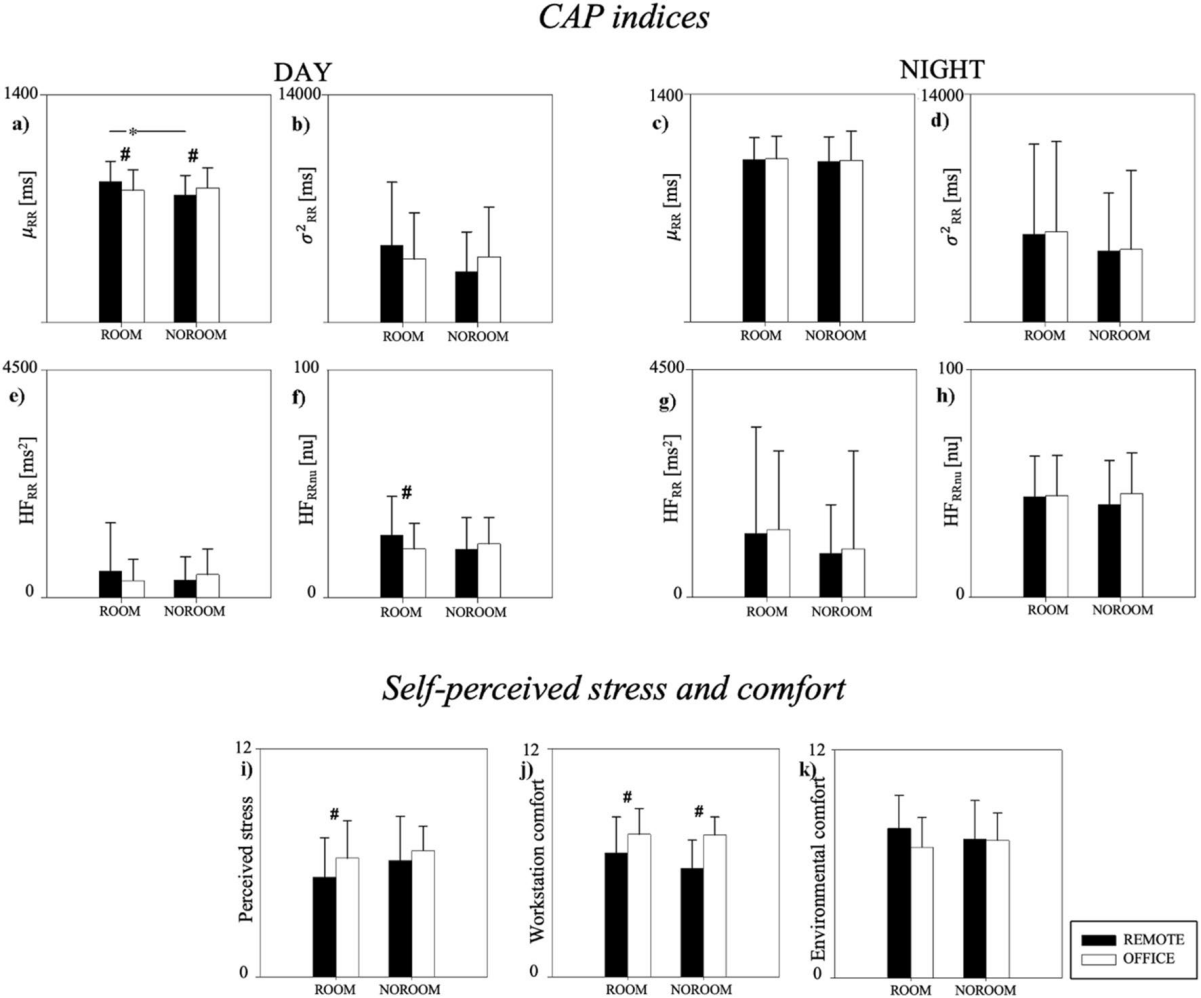
Comparison of CAP indices (panels (**a**–**h**)), self-perceived stress (panel (**i**)), environmental comfort (panel (**j**)) and workstation comfort (panel (**k**)) between REMOTE and OFFICE stratifying the population according to the presence (ROOM) or absence (NOROOM) of a dedicated work place while working from home. CAP indices were compared during daytime (DAY, panels (**a**,**b**,**e**,**f**)) and during night-time (NIGHT, panels (**c**,**d**,**g**,**h**)). Results are presented as mean ± standard deviation. *Indicates p < 0.05 DAY vs. NIGHT. ^#^Indicates p < 0.05 REMOTE vs. OFFICE.

To study the influence of the presence of dedicated workspace, the population was divided into two groups: ROOM group (n = 32, age 38 ± 11 years, 16 males and 16 females) and NOROOM group (n = 18, age 41 ± 9 years, 9 males and 9 females). The results are displayed in Fig. 4. During DAY, µ_RR_ was higher during REMOTE than OFFICE in the ROOM group. The opposite result was observed in the NOROOM group. In REMOTE, µ_RR_ was higher in the ROOM group compared to the NOROOM one. The diurnal HF_RRnu_ was higher in REMOTE compared to OFFICE in the ROOM group. No significant differences were detected for σ^2^_RR_ and HF_RR_ during DAY and µ_RR_, σ^2^_RR_, HF_RR_, and HF_RRnu_ during NIGHT in both ROOM and NOROOM groups. Subjects in the ROOM group perceived themselves as more stressed when in the OFFICE. The workstation comfort was better in OFFICE both for ROOM and NOROOM groups. No differences were noticed in the reported indoor environmental quality.

**Figure 5:**
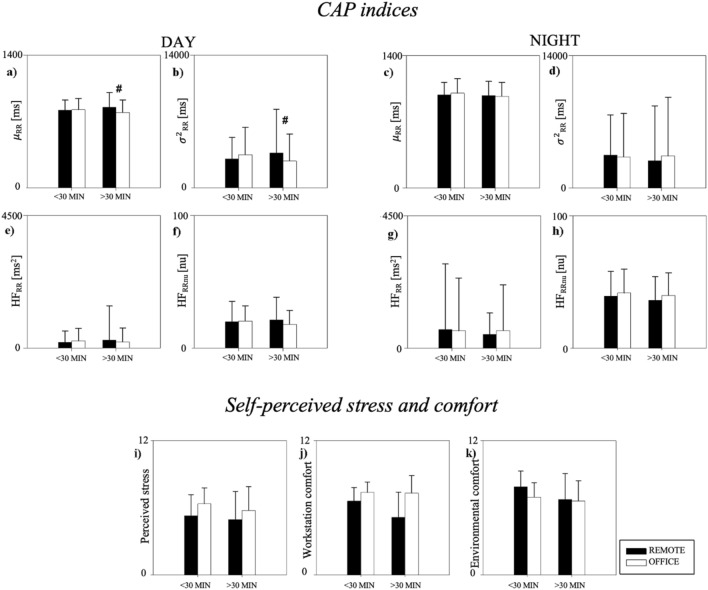
Comparison of CAP indices (panels (**a**–**h**)), self-perceived stress (panel (**i**)), environmental (panel (**j**)) and workstation comfort (panel (**k**)) between REMOTE and OFFICE stratifying the population according to the time for commuting. Office employees were classified in the group < 30 MIN, or > 30 MIN, if they spent less, or more, than 30 min to reach the workplace, respectively. CAP indices were compared during daytime (DAY, panels (**a**,**b**,**e**,**f**)) and during night-time (NIGHT, panels (**c**,**d**,**g**,**h**)). Results are presented as mean ± standard deviation. *Indicates p < 0.05 DAY vs. NIGHT. ^#^Indicates p < 0.05 REMOTE vs. OFFICE.

The comparisons of CAP indices, perceived stress, indoor environmental comfort, and workstation comfort between REMOTE and OFFICE splitting the population based on the commuting time are presented in Fig. 5. The population was divided into a group of subjects who reached the workplace in a relatively short time (< 30 MIN group, n = 28, age 38 ± 11 years, 13 males and 15 females) and a group of subjects who spent more than 30 min commuting (> 30 MIN group, n = 22, age 40 ± 11 years, 12 males and 10 females). During DAY, µ_RR_ and σ^2^_RR_ were higher during REMOTE than OFFICE in the > 30 MIN group. No further differences were found in the CAP indices, perceived stress, indoor environmental comfort, and workstation comfort.

## Discussion

To our knowledge, this is the first study comparing the effects of remote work and office work on cardiac autonomic parameters, perceived stress, environmental, and workstation comfort, their interrelationships, and relationships to objective factors related to individual or environmental conditions.

The present study highlighted several findings possibly interconnected in a complex environmental scenario. While the cardiac autonomic profile was similar during REMOTE and OFFICE days considering all participants as a single group, a different cardiac autonomic profile was identified during REMOTE in the subjects with cohabiting children, who were characterized by a lower cardiac vagal modulation during NIGHT compared to those without children. These findings could be driven by the higher engagement of workers with parenthood duties, dealing with the combination of the management of family life and work activities, which would affect the CAP towards a decreased cardiac vagal modulation. This evidence is in keeping with recent findings demonstrating that female healthcare professionals with pre-schoolers were characterized by lower cardiac vagal control compared to their nulliparous colleagues^23,24^.

Also, the results of the study suggested that routinely exceeding the theoretical number of scheduled hours could influence worker’s health. Indeed, subjects working for longer hours per day showed a decreased nocturnal cardiac vagal modulation both during REMOTE and OFFICE. Furthermore, overall significant differences were found regarding the degree of perceived stress (lower in REMOTE), environmental comfort (higher in REMOTE), and workstation comfort (lower in REMOTE). Somehow accordingly, during REMOTE workers with a dedicated workspace at home were characterized by a greater cardiac vagal modulation during DAY than those without a quiet workroom, and workers who spent a longer time commuting from home to the office had lower diurnal RR. Lastly, the sedentary habits and slept hours per night were not related to the working setting.

The above-mentioned results suggest that attention should be paid to evaluating the environmental conditions during both in-office and remote working, as the short and long-term effects of such conditions may influence the workers’ health. In fact, this study confirms that familiar and workplace factors seem to affect the balance in vagal cardiac control^4–9,38^. Indeed, the interesting results of the present study derived from the analysis of the relations between the state of the CAP and individual and environmental factors linked to the working conditions, which are not standardized in remote working with several interindividual differences. The main emerging conditions influencing the CAP were the presence of children at home, the unavailability of a dedicated workspace during remote working, and the number of working hours per day. These factors lead to an imbalanced CAP towards decreased cardiac vagal modulation. It is well known that a reduced vagal cardiac modulation and, indirectly, an inversely enhanced sympathetic one, represents a higher risk factor for cardiovascular disease development and a negative prognostic factor in several cardiac and non-cardiac diseases^39^.

Sleep is physiologically used as a period of cardiovascular relaxation and autonomic quiescence, as opposed to the daytime when the sympathovagal balance shifts towards an active mode^23,40^. The workers subject to working in somewhat uncomfortable conditions, whether due to the presence of little comfort, demanding children, or environmental pressures, could suffer from an imbalance in the CAP, which, over time, could represent a negative prognostic factor. Given the burden of cardiovascular diseases that account for approximately one-third of all deaths globally^41^, modifiable risk factors should be identified to ameliorate outcomes.

These results reinforce the recent literature^42,43^, showing some emerging psychological disturbances related to the overlapping of working commitments and private lives of workers, due to the overuse of smartphones or other electronic devices to connect with colleagues at any time. Such permanent engagement would add pressure to the workers possibly increasing distress and overall health risk. The remote working modality, blurring lines between working and domestic demands, introducing changes in familial relationships, adding job-related stress, and changing productivity, may add novel risk factors. The assessment of these risks is objectively difficult because of the number of individual and environmental variables; however, future research should try to better identify the objective risks to which employees could be exposed and take prevention measures.

The present study represents one of the first attempts to describe the impact of some personal and environmental factors on the CAP of healthy employees while working in the office or at home. Additional factors that could modify the CAP should be better studied, such as nutrition, workload and responsibility load, noise, heating, ventilation, and air quality conditions, personal comfort systems and number and age of the offspring using standardized scales^44,45^. Lastly, future research should then identify the cluster of workers who could benefit from remote working conditions and detect which factors most influence the workers’ health in this condition.

### Conclusion

These findings suggest the need for an adequate and exhaustive evaluation of workers’ health and working conditions to prevent long-term negative effects on their health. A full understanding of how working habits may change people’s lifestyles could provide important insights into the organization and management of office and remote work activities in the years to come, with the ultimate goal of improving employee well-being. In addition, it could aid cardiovascular disease prevention, as the observed altered cardiac vagal modulation in the employees could be associated with an increased risk for the development of cardiovascular and cardio-metabolic dysfunctions, as it happens for work-related stress. The winning approach should probably be a multidisciplinary monitoring program through the years, involving occupational medicine, cardiology, and psychology, to propose new evaluation protocols for office employees working in remote conditions to monitor their health.

### Limitations

The strengths of this study should be taken considering the presence of some limitations. Our protocol was conducted during the first year of the Covid-19 pandemic, a particular context associated with a big amount of sudden social changes, hence the comfort related to remote working would need to be re-evaluated in the future. Moreover, in our protocol, we considered the alternation between remote and in-office working within the same week. It could be of interest to investigate if different types of shifts would lead to the same results, and if repeated measures in different weeks would be replicable/reproducible over longer periods. In addition, the age of the enrolled subjects is not fully representative of the whole category of workers, then the external validity of these data should be evaluated according to age categories. The sample size is also limited, so the results should be confirmed in larger populations. Gender differences remain to be established, as in this study the sample size did not allow such analysis in subgroups. Lastly, in future research, environmental comfort should be deeply evaluated using cross-cultural validated scales such as the ASHRAE scale^45^, and it should be performed a more detailed and objective evaluation of the cofounding factors influencing the cardiac autonomic profile, the stress, and the workplace comfort in the two working conditions.

## Data Availability

Data are available upon reasonable request to the corresponding author.
